# The Harley Street Establishment for Invalid Gentlewomen

**Published:** 1887-04-16

**Authors:** 


### The Harley Street Establishment for Invalid Gentlewomen.

By a Lady Visitor.

There is a small home hospital in one of the streets leading out of Cavendish Square, to which I would much like to call the attention of the readers of this paper. I speak of the "Establishment for Invalid Gentlewoman," at 90, Harley Street. It has never been much advertised, the funds having been spent in maintaining its efficiency, but it is indeed quietly doing such a very good work, that it deserves to be better known and better supported.

Here, for the small weekly payment of from £1 1*s.* to £2 5*s.* 6*d.*, according to the bedroom accommodation necessary for their illness, the patients receive medical attendance and surgical aid, from men eminent in the different branches of their profession. The doctors give their valuable services gratis,—all honour to them! and some of the most difficult cases, necessitating serious operations, pass through the skilled hands of the surgeons, with marked success. The nursing is devotedly and conscientiously carried on by trained nurses, under the constant and active surpervision of the lady supertendent, herself a trained and experienced nurse. Those who are not themselves nurses cannot help wondering, when cases follow each other so rapidly, how each separate case seems to be the one special interest of those in charge.

The house is a large, handsome one, built in the last century, with its inner and outer hall and fine well staircase lighted from the top, the painted ceilings and carved mantelpieces giving to the lofty, well-proportioned rooms a look of completeness and refinement not often met with in more modern houses. On the ground-floor there is a dining-room for those patients well enough to leave their rooms. On the first-floor a good drawing-room, provided with plenty of sofas and easy chairs. The former drawing-room of the house is now turned into a ward to accommodate six patients; each compartment is curtained off, and fully furnished with all that is necessary for its occupant. These little rooms are generally allotted to those who are well enough to go about in the day, and who often prefer to pay £1 1*s.* a week, rather than the higher charges for the single bedrooms; but these, of course, in many cases are most desirable for patients. The bedrooms, many of them large, are all bright and comfortable in every respect.

The house is ordered throughout like a well-appointed home, where all alike enjoy privacy, care, and comfort. No expense is spared to provide anything that is deemed likely to facilitate the recovery of a patient. Game, fruit, and flowers are constantly sent as presents by the ladies of the committee.

Last year 121 patients were nursed in the house; of these, 36 required surgical treatment. Amongst the operations we may mention fourteen cases of tumour, four of which were cancers, and seven operations on the eye.

This house supplies a want much and widely felt in all parts of the country. There are none amongst us who do not know ladies with very small means, barely sufficient for the common needs of life; these can ill-afford the many things necessary in ordinary illness, but find it quite impossible to meet the strain when special medical or surgical treatment is required. How can small incomes bear the expenses of surgeons' fees, trained nurses, London lodgings, and the many costly appliances so often necessary in such cases? and who but those who have experienced it, can tell the relief it must be to many a sufferer to find all such cares and considerations taken off their hands, and to know that for a certain small fixed sum weekly, everything will be fully and amply provided, to give them as good a chance of recovery as could be afforded to the richest in the land? Some there are, who still, perhaps, feel a certain shrinking from the idea of a home or a hospital but these are daily becoming fewer, as the advantages of systematic treatment and trained nursing for serious and difficult cases are becoming more and more widely known and appreciated; so much so, that private hospitals are everywhere springing up to meet the requirements of wealthy patients.

The payments of the patients do not nearly cover the expenses, and carefully administered as the income has been, at the end of every year great and self-denying efforts on the part of the committee have always been necessary to enable the institution to start again without a balance on the wrong side.

Like other charitable works, this, too, has suffered from the "hard times" of which all complain, and increased subscriptions are most urgently needed, to enable the hospital to continue in its present efficient working order. Surely there are many who would help if they realised the great need there is for their assistance, and some, perhaps, who, shrinking with a nervous dread from themselves seeing illness and suffering, would be glad of an opportunity to give, for Christ's sake, some little aid to those who are working so indefatigably for those less fortunate than themselves. In order somewhat to increase the funds, one room in the house has of late been used for so-called paying patients, namely, anyone who is sufficiently well off to pay fully for all that she requires, and who in consequence could not be received there in any other way. This has been so far found to work very well, and many of the wealthier patients express themselves as truly grateful for the successful care which has been bestowed upon them.

Perhaps a few words may with advantage be added as to the name of this institution, namely, Establishment for Invalid Gentlewomen. It must be admitted that it sounds vague and unsatisfactory. "Hospital" for Gentlewomen would be a much better name, representing much more truly the scope of its work. The lady superintendent, 90, Harley Street, W., will gladly give any information about this "hospital."

### AN INCANTATION.

Ho! sick and weary! lay down the burden of life, enter and rest awhile;
The skill of man, the care of woman awaits you here;
Send your prayers upwards on the breath of flowers—
And bring down Faith, Hope, Patience, to aid those who tend, and you who suffer.—A. M. E.

